# Physical Modalities for the Treatment of Pain in Patients with Fibromyalgia

**DOI:** 10.31138/mjr.041124.pht

**Published:** 2025-03-31

**Authors:** Ilke Coskun Benlidayi

**Affiliations:** Cukurova University Faculty of Medicine, Department of Physical Medicine and Rehabilitation, Adana, Türkiye

**Keywords:** fibromyalgia, pain, physical therapy modalities, physical medicine and rehabilitation

## Abstract

Generalised pain is the major symptom in patients with fibromyalgia. The management of pain includes both pharmacological and non-pharmacological options. Exercise, meditative movement therapies and mindfulness-based stress reduction are examples of non-pharmacological treatments. Over the last decades, there is growing evidence regarding the role of physical modalities in the management of fibromyalgia-related pain. Physical modalities demonstrate their effects by using several energy types such as electrical, thermal, acoustic, or radiant energy. They may act through the alteration of blood flow, cellular activity, and nerve excitability. By reviewing the recent literature, the current article aimed to provide a comprehensive insight to the potential effects of physical modalities in the treatment of fibromyalgia-related pain. Evidence regarding the potential therapeutic role of transcutaneous electrical nerve stimulation, interferential current, therapeutic ultrasound, non-invasive brain stimulation techniques (e.g. transcranial direct current stimulation, transcranial magnetic stimulation), photobiomodulation therapy [e.g. Light Amplification by Stimulated Emission of Radiation (LASER)], the use of therapeutic cold (e.g. whole-body cryotherapy) was discussed.

## INTRODUCTION

Fibromyalgia is a rheumatic disease with multiple features including-but not limited to-generalised pain, fatigue, sleep disturbances and mood disorders.^1^ Widespread pain, which is the hallmark of fibromyalgia, interferes with the patient’s quality of life and general well-being. Thus, proper management of pain is essential in the treatment of fibromyalgia. The latest treatment recommendations by the European League Against Rheumatism (EULAR) includes both pharmacological and non-pharmacological approaches for the management of fibromyalgia. Moreover, EULAR endorses a graduated treatment plan focusing on non-pharmacological strategies at the beginning. Exercise is the cornerstone of non-pharmacological treatment. There are other non-pharmacological management options such as meditative movement therapies, mindfulness-based stress reduction and acupuncture.^2^ Physical modalities refer to a group of treatment options in which the therapeutic effect of physical forces such as heat/cold, light, sound, or electricity is used. They serve as potential and adjunctive treatment regimens in the management of pain.^3^ The present article aimed to provide an insight regarding the role of physical modalities in the treatment of fibromyalgia.

## SEARCH STRATEGY

**Figure 1.**
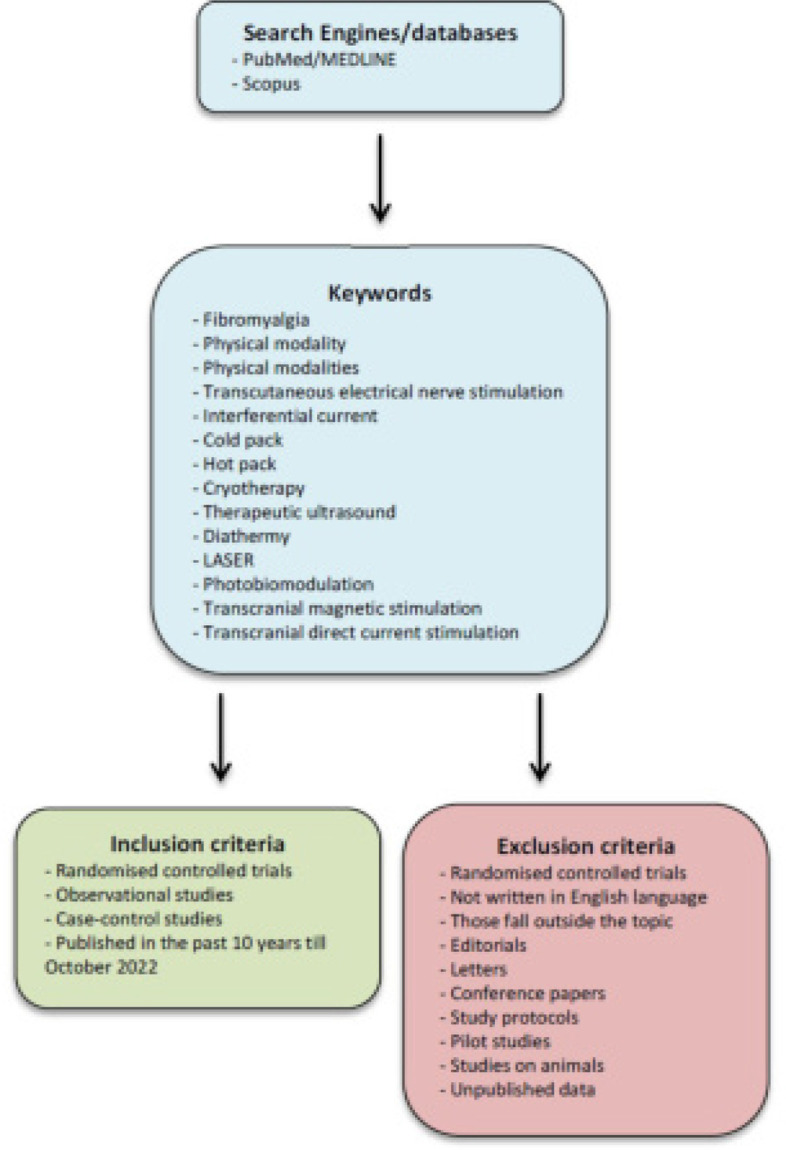
Flowchart of the search algorithm.

The current narrative review applied a search strategy recommended for narrative reviews.^4^ PubMed/MED-LINE and Scopus were searched for relevant articles by using the keywords “fibromyalgia”, “physical modality”, “physical modalities”, “transcutaneous electrical nerve stimulation”, “interferential current”, “cold pack”, “hot pack”, “cryotherapy”, “therapeutic ultrasound”, “diathermy”, “LASER”, “photobiomodulation”, “transcranial magnetic stimulation”, “transcranial direct current stimulation”. Randomised controlled trials, observational studies, and case-control studies published in the past 10 years till October 2022 were evaluated for eligibility. Exclusion criteria were i) articles that were not written in English language, ii) those fall outside the topic of the current review, iii) editorials, letters, conference papers, iv) study protocols, v) pilot studies, vi) studies on animals, and vii) unpublished data (**Figure 1**). The included articles’ reference lists were also evaluated for further relevant papers.

## WHAT ARE THE TYPES OF PHYSICAL MODALITIES?

**Figure 2.**
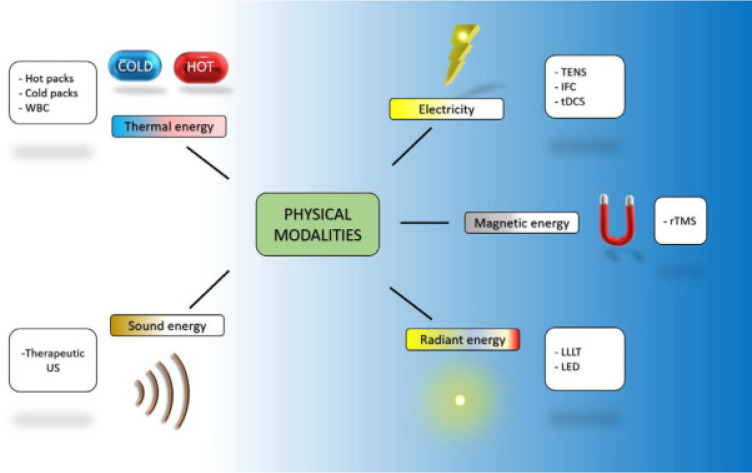
Physical modalities. Illustrated by the author. WBC: whole-body cryotherapy; TENS: transcutaneous electrical nerve stimulation; tDCS: transcranial direct current stimulation; rTMS: repetitive transcranial magnetic stimulation; US: ultrasound, IFC: interferential current; LLLT: low-level LASER therapy; LED: low-intensity light-emitting diode.

Physical modalities are a variety of therapeutic instruments, machines, and tools particularly used for the management of painful conditions/diseases of the musculoskeletal system. Their mechanism of action is primarily based on the transmission of energy to or through the patient.^3^ Electrotherapeutic modalities use electricity while physical agents involve thermal, acoustic, or radiant energy.^5^ Transcutaneous electrical nerve stimulation (TENS) and interferential current are examples of electrotherapeutic modalities. On the other hand, hot packs (superficial heat) and therapeutic ultrasound are examples of physical modalities using thermal and acoustic energy, respectively (**Figure 2**).

### How do physical modalities improve pain?

There are several mechanisms underlying the pain-relief effect of physical modalities:

Electrical physical modalities serve through the activation of pain gate mechanisms, transferring mechanical forces in order to change the physical properties of tissues, stimulation of cellular activity involving in healing and repair, alteration of blood flow and reduction of inflammation.^6^ Thermal physical agents induce vasodilatation, increase blood flow and metabolism, enhance extensibility and activate the transient receptor potential (TRP) channel, thereby reduce pain.^7^

Photobiomodulation therapy (PBMT) can induce cell proliferation and prevent cell death, thus can enhance tissue regeneration and the healing process. It also has anti-inflammatory and analgesic effects. The PBMT light is directly absorbed by the receptors of nerve cells. Following the absorption, the PBMT light increases the porosity of the nerve cell membrane and leads to the increased reabsorption of sodium ions, while the expulsion of potassium ions. The rebalance of the sodium-potassium pump within the nerve cell membrane prevents the nerve from reaching threshold. The mechanism underlying the analgesic effects of PBMT resembles the postsynaptic inhibition achieved by opioids.^8^

Therapeutic effectiveness of ultrasound is based on thermal and non-thermal effects. By its thermal effects, therapeutic ultrasound can cause changes in nerve conduction velocity, enzymatic activity, and contractile activity of skeletal muscles. It can also increase collagen tissue extensibility, local blood flow and pain threshold, and can reduce muscle spasm. On the other hand, non-thermal effects can lead to alteration in cell membrane activity, vascular wall permeability, and facilitation of soft tissue healing.^9^

### What is already known regarding the potential therapeutic role of physical modalities in fibromyalgia?

It is a well-known fact that exercising is the cornerstone of non-pharmacological management of fibromyalgia. Aerobic or mixed exercise regimens have been shown to improve pain intensity and health related quality of life in patients with fibromyalgia.^10,11^ In this regard, EULAR provided strong recommendations for exercising in the management of fibromyalgia.^2^ Yet, given the complex nature of fibromyalgia, other non-pharmacological options such as physical modalities may have potential value as adjuvant therapy options in management.

The effectiveness of several physical modalities in the management of fibromyalgia-related pain has been studied so far (**Table 1** and **Table 2**). Transcutaneous electrical nerve stimulation is one of the most studied modalities among patients with fibromyalgia.^12–27^ A Cochrane review by Johnson et al. evaluated 7 randomised controlled trials and 1 quasi-randomised trial of TENS treatment for pain related to fibromyalgia in 315 adult patients.^28^ The authors of the Cochrane review concluded that there was insufficient high-quality evidence to support or refute the use of TENS for patients with fibromyalgia.^28^ After then, several other studies assessed potential TENS-induced pain reduction in fibromyalgia. For instance, the Fibromyalgia Activity Study with TENS (FAST) study by Dailey et al., examined the effects of modulated frequency (2–125 Hz) TENS with highest tolerable intensity applied to the lumbar and cervicothoracic regions 2 hours/day for 4 weeks.^15^ Patients in the active TENS group exhibited greater improvement in movement-evoked pain, movement-evoked fatigue and global impression of change compared to those in placebo TENS or no TENS groups.^15^ Secondary analysis of data from FAST study revealed that the response to an initial 30-minute TENS treatment was a predictor of the response to longer-term TENS. Moreover, the results showed that TENS was safe and effective. Number needed to treat for pain and fatigue were 3.3 and 5.3, respectively. Number needed to harm for individual adverse events ranged between 20 and 100 for active-TENS when compared to either placebo-TENS or no-TENS treatments.^16^ Jamison et al., in their randomised controlled study, evaluated the effects of TENS in fibromyalgia.^14^ The results revealed modest treatment effects on pain, functional impairment and disease impact. Moreover, patients with higher pain sensitivity exhibited greater patient global impression of change by TENS than those with lower pain sensitivity. Wearable TENS appeared to be a safe treatment option for patients with fibromyalgia.^14^ It is of note to state that studies so far vary in the design (frequency, intensity, duration, treatment groups) and evaluation (outcome measures) of TENS treatments. As a cheap, easily-applied and relatively safe modality, TENS stands as a potential adjunctive treatment option for patients with fibromyalgia. Yet, adequately powered future studies with complete reporting of study designs and treatment interventions would be of value to improve our understanding in terms of the effects of TENS on fibromyalgia-related pain. The results of the studies involving the effects of TENS for fibromyalgia-related pain are provided in **Table 1**.

**Table 1.** Results of studies involving effects of transcutaneous electrical nerve stimulation, therapeutic ultrasound, interferential current, and photobiomodulation therapy for fibromyalgia-related pain.

**Study**	**Intervention(s)**	**Outcome measures**	**Results**
Dailey et al.^12^	-Active TENS -Placebo TENS -No TENS	Movement-evoked pain, NRS-resting pain, SF-36, fatigue, FIQR, BPI, Tampa Scale of Kinesiophobia	Active TENS was equally effective in terms of movement-evoked pain in patients regularly taking opioid medication and those not-regularly taking opioids.
Jamison et al.^13^	-Active wearable TENS -Sham wearable TENS	PGIC, FIQR, BPI, painDETECT, PDI, HADS, PCS	In the intention-to-treat population, FIQR total score, FIQR pain item, BPI Interference, and PDQ showed significant improvements for active TENS compared to sham TENS; yet, regarding PGIC, no difference was detected between groups at 3 months. In patients with higher pain sensitivity, PGIC was significantly greater for active TENS.
Jamison et al.^14^	-Active wearable TENS -Sham wearable TENS	PGIC, BPI, PDI, PCS, HADS, FIQR, painDETECT, perceived helpfulness	No differences in PGIC between groups. Patients with greater hypersensitivity benefited most from TENS.
Dailey et al.^15^	-Active TENS [modulated frequency (2–125 Hz), highest tolerable intensity] -Placebo TENS -No TENS	Movement-evoked pain and fatigue (6-minute walk test and 5-time sit-to-stand test; NRS), pain intensity and interference (BPI), resting pain and fatigue (NRS, MAF), Pain Self-Efficacy Questionnaire, PCS, FIQ, SF-36, fear of movement(Tampa Scale of Kinesiophobia), depression and anxiety (PROMIS short forms)	Greater reduction was observed in movement-evoked pain and fatigue by active TENS versus placebo TENS and versus no TENS.
Castro- Sánchez et al.^17^	-TENS (pulse width 200 µs, pulse frequency 100 Hz, burst frequency 2 Hz for 10 min for each pain dermatome) -Dry needling	Short Form pain scale of the McGill Pain Questionnaire, heart rate variability, galvanic skin response, SpO2 and photoplethysmography	Dry needling provided greater improvements across all dimensions of pain, higher difference for several parameters of heart rate variability and galvanic skin responses. No between-group differences were observed in spectral analysis of the photoplethysmography and SpO2.
Scaturro et al.^18^	-LASER + TENS+ Exercise -Exercise Unrelated control group disease: 10 patients with mechanical pain	FIQ, SF36, VAS pain, VAS fatigue, sleep dysfunction	The combined treatment provided significant improvement in pain, fatigue, and FIQ; yet not in the SF36.
Yüksel et al.^19^	-TENS -Acupuncture	Quantitative EEG changes, Pain	TENS resulted an increase in the alpha power of the left anterior region. Acupuncture resulted an increase in the alpha power of the right and left posterior regions. Both treatments improved pain scores.
Thieme et al.^20^	-OT-TENS -SET -AE-BRT	Manual Tender Point Survey, MPI, NRS-pain, BRS, treatment expectation ratings and satisfaction ratings	SET provided greater reduction in pain and pain interference.
Dailey et al.^21^	-Active TENS -Placebo TENS -No TENS	Pain and fatigue at rest (VAS), pain and fatigue in movement (VAS), Conditioned Pain Modulation, 6-m walk test, 5-time sit- to-stand test), pressure pain thresholds, range of motion, single-leg stance	Compared to placebo TENS and no TENS; Active TENS improved pain and fatigue with movement; increased pressure pain thresholds; provided a significantly stronger conditioned pain modulation. Functional tasks showed no change with TENS.
Lauretti et al.^22^	-Single-TENS (1 active and 1 placebo TENS) -Double-TENS (2 active TENS devices) -Placebo TENS (no stimulus)	VAS pain, consumption of analgesics, quality of sleep, fatigue	Pain relief, improvement in quality of sleep and disposition were double-TENS > single-TENS > placebo TENS
Carbonairo et al.^23^	-Education + AE + static stretching + TENS -Education + AE + static stretching	VAS pain, tender points pain threshold, FIQ	Change in pain intensity was higher in patients received TENS. Patients who did not receive TENS showed worsening of the pain threshold.
Mutlu et al.^24^	-Supervised exercise + TENS -Supervised exercise	Tender point count, myalgic pain score, FIQ, SF-36	The improvement in myalgic pain was higher in the exercise+TENS group at the 3^rd^ week, yet not different at the 12^th^week. The improvement in other outcomes was not different between groups.
Moretti et al.^80^	US+IFC (Once a week vs twice a week)	VAS, FIQ, Post Sleep Inventory, Tender point count	Both groups showed improvement in outcome measures. No significant between-group differences.
Germano et al.^71^	-LLLT (functional exercise program associated with active phototherapy: 40 to 60’, 3 times a week for 8 weeks, 808 nm, 100 mW, 4 J, 142.85 J/cm^2^ per point, applied bilaterally to different points of the quadriceps, hamstrings, and triceps sural muscles immediately after each exercise session ) -Placebo (functional exercise program associated with placebo phototherapy)	Pain sites, pain intensity, pain threshold, balance, functional tests, muscle flexibility, isokinetic variables, depression, QoL	No significant differences were observed between groups.
da Silva et al.^72^	-Phototherapy irradiation (11 locations, employing a cluster with 9 diodes (1 super-pulsed infrared 905 nm, 4 LEDs of 640 nm, and 4 LEDs of 875 nm, 39.3 J per location) -Phototherapy+exercise training -Exercise training and phototherapy placebo -Control	Algometry, VAS-pain, FIQ and Research Diagnostic Criteria (RDC) instruments, SF-36	LEDs and exercise training improved the pain threshold. Combined therapy provided a more substantial effect.
Vayvay et al.^73^	-Low-level Ga-AS LASER, 100–240 v, 50–60 hz 75 VA, 2 J/cm^2^, 40 mw, wavelength: 850 nm, 3’ each on painful points+exercise -Placebo LASER+exercise -Taping application+exercise	VAS-pain, trunk flexibility, FIQ, SF-36, BDI	In the LASER group, improvement in pain severity in activity, anxiety level, general health status, QoL was observed. Trunk flexibility improved in the taping group. Pain severity at night and functional status improved after all interventions.
Nakajima et al.^74^	Bilateral xenon light irradiation (0.38–1.1 μm) around the stellate ganglion, 15’	VAS-pain, FIQ	Xenon light irradiation of the stellate ganglion provided significant improvement in pain scores in patients with a higher score in the FIQ.
Ruaro et al.^75^	-LLLT (GaAlAs LASER, 20 mW, 670nm, 4 J/cm^2^, 7sx4 for 18 tender points -Sham	FIQ, McGill Pain Questionnaire, VAS, tender point count	Although the number of tender points reduced in both groups, only LLLT provided significant improvements in FIQ, McGill Pain Questionnaire and VAS.
Panton et al.^76^	-Class IV LASER (twice weekly sessions for 4 weeks, continuous-wave, dual-wavelength LASER with 20% 810 nm, and 80% 980 nm at 10 W ) and heat therapy -Sham treatment and heat therapy	Myalgic score, FIQ, CS-PFP test	Compared to the sham group, LASER heat therapy significantly improved pain and FM impact measured by the FIQ and upper body flexibility measured by the CS-PFP test.
Klemm et al.^85^	Whole-body cryotherapy (6 sessions (−130 °C in 6 weeks)	VAS-pain after 6 sessions, FIQ, VAS-pain after 3 sessions and 3 months after discontinuation of therapy, cytokine levels (IL-1, IL-6, IL-10, TNF-α), patients’ opinions on the satisfaction, effectiveness and significance of whole-body cryotherapy	Whole-body cryotherapy provided a significant reduction in pain and disease activity after 3 and 6 sessions. Patients with FM revealed significantly different response of IL-1, IL-6 and Il-10 to whole-body cryotherapy.
Rivera et al.^82^	Cryotherapy (cryosauna cabin) vs control	VAS-pain, FIQ, ICAF	Following the first period, change in pain, FIQ and ICAF scores were significantly greater in the whole-body cryotherapy group.
Yilmaz et al.^81^	Local cold application (cold gel pack, 10’ to one trapezius muscle)	VAS-pain	Local cold application to the trapezius muscle providedsignificant improvement in pain.

FM: fibromyalgia; RTO: regularly taking opioids; MAF: multidimensional assessment of fatigue; US: ultrasound; IFC: interferential current; VAS: Visual Analogue Scale; FIQ: Fibromyalgia Impact Questionnaire; FIQR: Fibromyalgia Impact Questionnaire Revised; PGIC: Patient Global Impressionof Change; BPI: Brief Pain Inventory; PDQ: painDETECT questionnaire; PDI: Pain Disability Inventory; PCS: Pain Catastrophizing Scale; PROMIS: Patient-Reported Outcomes Measurement Information System; HADS: Hospital Anxiety and Depression Scale; painDETECT: Pain Detect Neuropathic Pain Questionnaire; SpO2: galvanic response and oxygen saturation; LASER: Light amplification by stimulated emission of radiation; SF-36: Short Form-36; EEG: electroencephalography; OT: Operant treatment; BRS: baroreflex sensitivity; AE: aerobic exercise; SET: systolic extinction training; MPI: Multidimensional Pain Inventory; NRS: numeric rating scale; LLLT: low-level laser therapy; IL: interleukin; TNF-α: tumor necrosis factor α; ICAF: Combined Index of Severity of Fibromyalgia; LEDs: light-emitting diodes; CS-PFP: Continuous Scale Physical Functional Performance.

**Table 2.** Results of studies involving the effects transcranial direct/alternating current stimulation, transcranial magnetic stimulation, and occipital nerve stimulation for fibromyalgia-related pain.

**Study**	**Intervention(s)**	**Stimulation area/duration**	**Outcome measures**	**Results**
Paula et al.^29^	-LDN+tDCS -LDN+tDCS Sham -Placebo+tDCS -Placebo+tDCS Sham	M1, 20’	VAS-pain, PCS, STAI, FIQ, BDI-II, PCP:S, PPT, CPM, BDNF serum level	The LDN+tDCS group experienced improvement in pain frequency, intensity, effect of pain on activities and emotions.
Arroyo-Fernández et al.^30^	-Active tDCS+exercise -Sham tDCS+exercise -No-intervention	5 sessions over 2 weeks	Main outcomes were pain intensity and referred pain area following suprathreshold pressure stimulation	Active tDCS group experienced further improvement in pain intensity. Compared to no-intervention, active and sham tDCS provided improvement in health status, pain catastrophising, and depression
Samartin-Veiga et al.^31^	-tDCS on left M1 -tDCS on left DLPFC -tDCS on left OIC -Sham tDCS	15 sessions of 20’	SF-36, FIQ-R	Although all groups showed improvement in QoL and symptoms’ impact, no group effect or group treatment interaction was observed.
Samartin-Veiga et al.^32^	-tDCS on left M1 -tDCS on left DLPFC -tDCS on left OIC -Sham tDCS	15 sessions of 20’	Pain intensity, fatigue, mood, cognitive and sleep disorders, PPT	Irrespective of the group, patients experienced significant improvement in clinical pain and for fatigue, cognitive and sleep disturbances, and experimental pain. All active tDCS groups revealed a significantly greater improvement in anxiety and depression when compared to sham tDCS.
Caumo et al.^33^	-Home-based anodal tDCS -Sham tDCS	Bifrontal, with anodal on the left DLPFC for 20’	PCS, Profile of Chronic Pain: Screen, depressive symptoms, sleep quality, heat pain threshold, heat pain tolerance, serum BDNF	tDCS provided reduction in PCS scores by 51.38% when compared to 26.96% observed by sham-tDCS. Profile of Chronic Pain: Screen total scores reduced by 31.43% in the active group, compared to 19.15% in the sham group. tDCS improved sleep quality and depressive symptoms; increased heat pain tolerance.
Matias et al.^34^	-tDCS associated with functional exercise -Sham-tDCS associated with functional exercise	Left motor cortex, 5 consecutive days during the first week of intervention	Pain intensity, functional performance, QoL, psychological symptoms	There were no significant between-group differences in terms of the improvement in outcome measures.
Kang et al.^35^	tDCS	20’ on 5 consecutive days	VAS-pain, FIQ, BPI, BFI, BDI, STAI, MOS-SS	46 patients experienced improvements in VAS pain scores (on days 6, 13, 36), in FIQ (on day 13), in BDI (on days 6 and 36), and in BFI (on days 6 and 13). No significant improvement was observed in STAI-I, STAI-II, and MOS-SS scores.
Brietzke et al.^36^	-Home-based anodal tDCS -Sham tDCS	Over the left DLPFC for 30’	VAS-pain, disability related to pain measured by Profile of Chronic Pain: Screen, analgesic use, psychological symptoms, sleep quality, PPT, heat pain threshold	Active tDCS improved cumulative pain scores by 45.65%, following the first 20 sessions. Active tDCS improved pain scores by 62.06% after 60 sessions. Active tDCS reduced the risk for analgesic use in 55%.
Khedr et al.^37^	-Real tDCS -Sham tDCS	Over left M1, 10 sessions	WPI, SS, VAS-pain, pain threshold, HAM-D and HAM-A, serum beta-endorphin level	The real tDCS group experienced higher improvement in WPI, SS, VAS, pain threshold, HAM-A, HAM-D. Serum beta-endorphin level was negatively correlated to the changes in different rating scales of pain and mood.
Silva et al.^38^	-Active tDCS -Sham tDCS	Over the DLPFC, 20’	Attention Network Test, heat pain threshold, Heat pain tolerance	Active tDCS provided improvement in the orienting and executive attention networks, with no effect on alertness. Active tDCS increased HPTh when compared to sham tDCS.
Mendonca et al.^39^	-tDCS + AE -AE only -tDCS only	Over M1, 5 days of stimulation for 20’	Pain intensity, anxiety, QoL, mood, PPT, cortical plasticity	In terms of pain intensity, tDCS/AE was superior to AE alone. There was significant difference between groups in terms of anxiety and mood levels, with tDCS/AE treatment provided the greatest response. In terms of responses in motor cortex plasticity, there was no difference among groups.
Cummiford et al.^40^	5 days of real tDCS and 5 days of sham tDCS	Over left M1	Resting state functional connectivity	There may be a placebo response to both sham and real tDCS. Yet, real tDCS caused distinct changes in functional connectivity that lasted beyond the treatment period. Stronger baseline functional connectivity between M1- ventral lateral thalamus, primary somatosensory cortices-anterior insula, and ventral lateral thalamus-periaqueductal gray predicted greater analgesia following sham and real tDCS. tDCS over M1 might produce analgesia by altering thalamic connectivity.
Castillo-Saavedra et al.^41^	HD-tDCS	Over left M1, 20’ per session	VAS-pain, pain diary, FIQ, BDI, Semmes-Weinstein Monofilament measurement, PPT, Neurophysiologic Outcomes: Contact Heat- Evoked Potentials	Half of the patients experienced clinically significant benefit of a 50% pain reduction. The median number of HD-tDCS sessions to reach clinically meaningful outcomes was estimated as 15.
Foerster et al.^42^	Sham tDCS (5 consecutive days), a 7-day washout, followed by active tDCS (5 consecutive days)	Over left M1	Proton magnetic resonance spectroscopy	Pain scores showed a significant decrease between the baseline and active tDCS time points. Following active tDCS, compared to sham tDCS, glutamate + glutamine levels (Glx) in the anterior cingulate were significantly lower. Baseline anterior cingulate Glx levels showed correlation with the improvement in pain score.
Fagerlund et al.^43^	-Active tDCS -Sham tDCS	Over M1, 5 consecutive sessions for 20’	NRS for pain intensity, pain unpleasantness, stress, anxiety; FIQ; HADS; The SCL-90R; SF36v2	Active tDCS provided a small but significant improvement in pain when compared to sham tDCS. Yet, the results are unlikely to reflect clinically important changes. Daily functioning improved by active tDCS group compared with the sham tDCS.
Villamar et al.^44^	Anodal, cathodal, and sham HD-tDCS	Left M1, single 20-minute sessions	VNS for pain, VNS for anxiety, Adapted QOL Scale, BDI-II, Semmes-Weinstein monofilaments for pain and mechanical detection threshold, PPT, diffuse noxious inhibitory controls	Both active stimulation conditions provided significant reduction in overall perceived pain (which occurred immediately after cathodal HD-tDCS) when compared to sham. Active anodal HD-tDCS induced a significant bilateral increase in mechanical detection thresholds.
Gómez-Arguelles et al.^46^	Low frequency (8 Hz) square wave with a picoTesla magnetic field	The insula, DLPFC Cingulum 20 min	Magnetoencephalography, FIQ, Pain threshold	TMS provided an increase in alpha brain oscillatory activity particularly in the left DLPFS and an improvement in FIQ.
Pareja et al.^47^	-Standard pharmacological treatment -Standard pharmacological treatment + LIMS	8 sessions of 20’ long, once a week	WPI, SS score, FIQ	Significant improvement was observed in WPI, SS score and FIQ from the second week following the last session. These beneficial effects were maintained throughout the 24 weeks of follow-up after the last intervention.
Argaman et al.^48^	-Real rTMS and Sham rTMS	M1, 10 daily treatments over 2 weeks, with a washout period in between	Functional connectivity FM-related symptomology	Only the real rTMS provided reductions in FM-related symptomology The changes showed correlation with resting-state functional connectivity in brain areas related to pain processing and modulation.
Izquierdo-Alventosa et al.^49^	-Physical exercise -High frequency TMS -Control group	Over M1, 5 sessions of 20’ per week for 2 weeks	VAS-pain, PPT, FIQR, 6-Minute Walking Test, CR-10 Borg scale for induced fatigue, 4-meter gait speed test, 5-repetition sit-to-stand test, HADS, BDI-II, Perceived Stress Scale-10, Satisfaction with Life Scale	TMS provided significant improvement in all studied variables-except for satisfaction. Physical exercise provided improvement in average PPT, perceived overall impact of FM and total score, endurance and functional capacity, velocity and power, anxiety, depression, and stress. Controls revealed no improvements in any outcome measures.
Forogh et al.^50^	- High-frequency rTMS - Anodal tDCS	Over the left DLPFC, 3 sessions over 1 week.	VAS-pain, FIQR, Depression Anxiety Stress Scale-21 Item	The behavior of two treatment groups differed regarding changes in VAS-pain in favor of the rTMS group. However, time-group interaction effect on DASS-21 and FIQR was not significant. rTMS provided at least a 30% reduction of VAS in 66.6% of patients, while 26.6% of patients in tDCS group experienced this improvement.
Guinot et al.^51^	-Active rTMS +multicomponent therapy - Sham rTMS +multicomponent therapy -Multicomponent therapy alone	Over M1; induction phase: 2 weeks, 5 sessions/week, followed by maintenance phase: 12 weeks, 2 sessions on week 3 (the first week of exercise training), then 1 session/week on weeks 4, 6, 9 and 13	Weekly mean self-reported level of VAS-pain, FIQ, BDI, PSQI, PCS, cardiorespiratory fitness, cardiac autonomic adaptations	There was no between-group difference in terms of the reduction of the weekly mean of pain reported daily. rTMS added to multicomponent therapy did not improve pain.
Bilir et al.^52^	-Active rTMS -Sham rTMS	Over the left DLPFC; induction phase: 10 sessions daily, 5 days per week for 2 weeks followed by maintenance phase: 4 sessions weekly, 1 day per week for 4 weeks	VAS-pain, FIQ, Fatigue Severity Scale, HADS, Addenbrooke’s cognitive examination-last revised version, VAS-stiffness	VAS-pain, Fatigue Severity Scale and HADS scores did not differ within or between groups over time. rTMS provided significant improvement in VAS-stiffness and FIQ at the 2nd week. Yet, no significant between-group difference was detected.
Tanwar et al.^53^	-Real rTMS -Sham rTMS	Over the right DLPFC, 5 consecutive days per week for 4 weeks	Numerical Pain Rating Scale, MPQ, Hamilton Depression Rating Scale, Hamilton Anxiety Rating Scale, WHOQOL-Quality of Life-BREF questionnaire, nociceptive flexion reflex, pain modulation, estimation of oxidative stress markers	Real-rTMS provided significant improvement in average pain ratings and associated symptoms. The improvements lasted up to 6 months. Sham-rTMS group provided no significant change in pain ratings.
Altas et al.^54^	-High frequency rTMS to left M1 - High frequency rTMS to left DLPFC -Sham rTMS	15 sessions over 3 weeks, 10 Hz	VAS, FIQ, Fatigue Severity Scale, SF-36, BDI	Active rTMS provided greater improvement in depression, physical functioning, physical role functioning, and general health perceptions. Active rTMS over M1 provided greater improvement in VAS pain compared to sham rTMS. Active rTMS over DLPFC provided greater change in physical role functioning compared to active rTMS over M1. An improvement in emotional functioning was observed only by active rTMS over M1.
Abd Elghany et al.^55^	-rTMS -Prolotherapy	rTMS sessions every other day for one month	VAS-pain, BDI, FIQ revised, cortical auditory evoked potentials, number of tender points	In the prolotherapy group compared to rTMS group, improvement in VAS-pain was remarked; difference in number of tender points and revised FIQ score were significant immediately after treatment and one month later. BID and cortical auditory evoked potentials showed better improvement in the rTMS group compared to the prolotherapy group.
Fitzgibbon et al.^56^	-Active rTMS -Sham rTMS	Left-hemisphere DLPFC, daily sessions over 4 weeks	SF-MPQ, BPI short form, NRS pain intensity and pain unpleasantness, SF-36v2, FIQ, Multidimensional Fatigue Inventory-20, PCS, BDI-II, BAI, Patients’ Global Impression of Change scale	By active rTMS, physical fatigue and general fatigue improved significantly. The active group had 2.84 times more chance to experience a minimum 30% improvement in pain intensity. Yet, no group difference for the primary outcome measures was observed.
Tzabazis et al.^57^	rTMS	Different configurations of rTMS, 20 daily sessions of 30’ over 4 weeks were applied to the FM group	BPI, FIQ, BDI-II, NRS	In patients with fibromyalgia, rTMS, operated at 10 Hz, provided 43% reduction in NRS pain over last 24 hours. This improvement was maintained for at least 4 weeks following the final session.
Maestú et al.^58^	-Very low intensity TMS -Sham TMS	One session (20’) per week for 8 weeks.	PPT, Blood serotonin level, VAS for ability to perform daily activities, perceived chronic pain intensity, fatigue, anxiety, depression, sleep quality, and severity of headaches	When compared to controls, active group revealed significant improvement in somatosensory pain thresholds, ability to perform daily activities, perceived chronic pain and sleep quality. Yet, scores of depression, fatigue, severity of headaches, as well as serotonin levels were not different between two groups.
Lee et al.^59^	-Low-frequency (1 Hz) rTMS to right DLPC -High-frequency (10 Hz) rTMS to left M1 -Sham stimulation	10 consecutive sessions	Number of tender points, FIQ, VAS-pain, BDI	Immediately after low-frequency rTMS, VAS-pain and FIQ scores decreased significantly. One month after low-frequency rTMS, BDI scores decreased significantly from baseline. Immediately after high-frequency rTMS, VAS-pain and BDI scores decreased significantly.
Baudic et al.^60^	-Active rTMS -Sham rTMS	Left M1, 14 stimulation sessions over 21 weeks	BPI, MOS-SF-12, HADS	No between-group difference in overall neuropsychological performance was detected.
Lin et al.^61^	-Active HD-tACS -Sham stimulation	Left M1 10 sessions in 2 weeks	NRS-pain intensity, FIQ, BAI and BDI-II, PSQI, PPT, total Tau and Beta-Amyloid 1–42	Although improvement in FIQ was detected after HD-tACS, no significant difference was observed in NRS and FIQ scores compared to sham stimulation. Although most adverse events were mild, one patient in HD-tACS group attempted suicide during the study.
Ahmed et al.^65^	-Active occipital nerve field stimulation -Device turned-off	Implanted subcutaneous electrode, sub-sensory threshold stimulation for 2 weeks	FIQ, Pain Vigilance and Awareness Questionnaire, PCS, NRS, H_2_^15^O PET scans, EEG data	Active occipital nerve stimulation led to an activation in the DLPFC. EEG data revealed increased activity in the descending pain pathway. Occipital nerve stimulation was shown to exert its effects by the activation of the descending pain inhibitory pathway and the lateral pain pathway.
Yoo et al.^66^	-Control with sham occipital stimulation -tDCS on the occipital nerve (ON) only -tDCS on bilateral DLPFC before occipital stimulation	8 sessions for 4 weeks, the sessions were 3 days apart from each other	FIQ, BDI, NRS-pain	Adding bifrontal tDCS to ONS has no added benefit in improving outcomes.
To et al.^67^	-tDCS over C2 area -tDCS over DLPFC -Sham tDCS	Over C2 area or DLPFC, 8 sessions: two times a week for 4 weeks	NRS, PCS, Modified Fatigue Impact Scale	tDCS to C2 area provided significant improvement in pain, but not in fatigue. tDCS over DLPFC significantly improved pain and fatigue.
Ridder and Vanneste^68^	-Occipital nerve field tDCS and Sham tDCS	Over C2 area; 1 consecutive week of sham tDCS and 1 week of active tDCS with a 2-week washout between the sham and active tDCS	NRS, FIQ, EEG data	Active occipital nerve field tDCS (but not sham protocol) improved dysfunctional effective connectivity from the pregenual anterior cingulate cortex to the dorsal anterior cingulate cortex.
Plazier et al.^69^	C2 nerve field stimulation	Implanted subcutaneous electrode in the C2 dermatoma; different doses of stimulation for 2 weeks	FIQ, Pain Vigilance and Awareness Questionnaire, PCS, tender point examination, NRS for pain and QoL, overall satisfaction, QoL, and overall symptom relief, BDI, Modified Fatigue Impact Scale, PSQI	Patients experienced an overall decrease of 36% and 33% on FIQ and pain, respectively. Daily life activities and quality improved by 42% during the first 6 weeks. The results maintained at 6 months follow up. Higher amplitudes seemed to provide better outcomes.
Plazierlaser et al.^70^	Greater occipital nerve stimulation	Implanted subcutaneous occipital nerve stimulator	FIQ, pain threshold, Laser-Evoked Potentials recordings	Despite a significant increase in N2P2 latencies (particularly at the Pz electrode), the amplitudes of the LEP recordings were not modified by occipital nerve stimulation.

FM: fibromyalgia; tDCS: transcranial direct current stimulation; HD-tACS: high-definition transcranial alternating current stimulation; M1: Primary motor cortex; NRS: numeric rating scale; FIQ: fibromyalgia impact questionnaire; BAI: Beck Anxiety Inventory; BDI-II: Beck Depression Inventory Second Edition; PSQI: Pittsburgh Sleep Quality Index; PPT: pressure pain threshold; DLPFC: dorsolateral prefrontal cortex; LDN: low-dose naltrexone; VAS: Visual Analog Scale; PCS: Pain Catastrophizing Scale; STAI: State-Trait Anxiety Inventory; PCP:S: Profile of Chronic Pain Scale; CPM: Conditioned Pain Modulation; SF-36: Short form 36; OIC: operculo-insular cortex; BPI: Brief Pain Inventory; BFI: Brief Fatigue Inventory; MOS-SS: Medical Outcomes Study Sleep Scale; ONS: occipital nerve stimulation; WPI: widespread pain index; SS: symptom severity of fibromyalgia; HAM-D: Hamilton depression scale; HAM-A: Hamilton anxiety scale; AE: aerobic exercise; HD-tDCS: high-definition transcranial direct current stimulation; SCL-90R: The Symptom Checklist-90-Revised; VNS: visual numbering scale; rTMS: repetitive transcranial magnetic stimulation; SF-MPQ: Short-Form McGill Pain Questionnaire; MOS-SF-12: Medical Outcomes Study Short Form 12; BNDF: brain-derived-neurotrophic-factor; LIMS: low-intensity magnetic stimulation; EEG: electroencephalography; PET: positron emission tomography.

Non-invasive brain stimulation techniques have been widely studied in fibromyalgia. Non-invasive brain stimulation techniques include transcranial direct current stimulation (tDCS),^29–45^ transcranial magnetic stimulation (TMS),^46–60^ high-definition transcranial alternating current stimulation (HD-tACS),^61^ cranial electrotherapy stimulation, transcranial random noise stimulation and reduced impedance non-invasive cortical electrostimulation.^62^ These stimulation techniques alter the excitability of the certain areas of the brain. The most commonly studied application areas are the primary motor cortex (M1) and the dorsolateral prefrontal cortex.^63^ A Cochrane review by O’Connell et al. concluded that, with very low-quality evidence, single doses of high-frequency rTMS of the motor cortex and tDCS may provide short-term benefits on chronic pain and quality of life among patients with fibromyalgia. The authors could not obtain similar benefit regarding low-frequency rTMS, rTMS applied to the dorsolateral pre-frontal cortex and cranial electrotherapy stimulation.^62^ After then, research on that topic has continued. For instance, Pareja et al. investigated the effects of transcranial low-intensity magnetic stimulation in women with fibromyalgia.^47^ After eight weeks of treatment, significant improvement in diagnostic variables were obtained. Moreover, the beneficial effects maintained up to 24 weeks after the last treatment session.47 Gomez-Arguelles et al. also reported favourable results by using low intensity TMS.^46^ Argaman et al. reported an association between the functional alterations of brain areas playing role in the experience of chronic pain and the acute clinical effects of rTMS of the motor cortex.^48^ Nevertheless, high-frequency rTMS to the left dorsolateral prefrontal cortex did not provide benefits on fibromyalgia-related symptoms.^52^

A randomised controlled trial by Izquierdo-Alventosa et al. investigated the effects of high-frequency TMS and physical exercise in women with fibromyalgia. Results showed similar benefits of TMS and exercise for physical status, whilst TMS provided more improvement than exercise for emotional status.^49^ Guinot et al. compared the effects of rTMS (high-frequency, primary motor cortex M1) and sham rTMS with a combination of 12-week multicomponent therapy.^51^ They found no significant difference between groups in terms of weekly mean of pain reported daily.^51^ Izquierdo-Alventosa found that TMS provided similar improvement in physical status when compared to low-intensity physical exercise program. However, in terms of emotional status, TMS provided greater benefit.^49^ A recent systematic review and meta-analysis investigating 7 studies with 217 patients concluded that 10-Hz rTMS can significantly improve pain and quality of life in patients with fibromyalgia.^64^ Moreover, a subgroup analysis based on stimulation at the primary motor cortex and dorsolateral prefrontal cortex revealed no significant difference.^64^ Forogh et al. performed a comparative analysis of rTMS and tDCS. Although both modalities appeared as safe treatment options, rTMS over dorsolateral prefrontal cortex provided greater and longer pain relief effect.^50^

Home-based electrical stimulation has also been studied recently.^33,36^ A randomised, double-blind, sham-controlled study showed that home-based bifrontal tDCS with a-tDCS on the left dorsolateral prefrontal cortex provided a decrease in rumination/magnification of pain catastrophising and improvement in the disability for daily activities due to fibromyalgia-related symptoms. Data derived from the trial supported the feasibility of a self-applied home-based version of tDCS.^33^ Another type of stimulation attracted attention in fibromyalgia is the occipital nerve stimulation, which can be performed by tDCS or implanted electrode.^65–70^ The results of the studies involving the effects of tDCS, TMS, HD-tACS, occipital nerve stimulation for fibromyalgia-related pain are provided in **Table 2**.

Light has been used as a therapeutic option in several musculoskeletal disorders. Over the lats decade, the potential effects of light have also been studied for fibromyalgia-related pain (**Table 1**).^71–76^ Photobiomodulation therapy is a non-thermal and non-ionising light therapy, which is applied in the form of Light Amplification by Stimulated Emission of Radiation (LASER) and red and/or near infrared low-intensity light-emitting diodes (LEDs).^8^ LASER, which is usually applied on tender points may provide some benefits in terms of pain relief.^63^ Ruaro et al. showed the beneficial effects of low-level laser therapy (LLLT) in pain relief among patients with fibromyalgia.^75^ Panton et al. also obtained favourable results; Class IV LASER therapy provided benefits in improving pain and upper body range of motion.^76^ There are studies evaluated the effects of LASER in orofacial pain or temporomandibular pain in patients with fibromyalgia.^77,78^ A randomised controlled trial by Molina-Torres et al. evaluated the effectiveness of LASER therapy applied to the tender points and of an occlusal stabilisation splint in patients with temporomandibular disorders and fibromyalgia.^78^ Both appeared as alternative therapeutic options for improving pain and the clicking sound for temporomandibular disorders.^78^ From a different point of view, add-on LLLT to exercise is used in healthy people to increase muscle performance and to avoid exercise-induced fatigue. However, there is conflicting data regarding the patients with fibromyalgia.^63^ In a double-blind, placebo-controlled randomised clinical trial evaluating the effects of LLLT combined to an exercise program in patients with fibromyalgia, LLLT was performed onto quadriceps, hamstrings and triceps sural muscles for 40–60 minutes, 3 times/week for 8 weeks immediately after the exercise. Low-level laser therapy failed to further improve the beneficial effects of functional exercise.^71^ Dos Santos et al. also reported that peripheral muscle strength and resistance did not show improvement following acute LLLT application.^79^

With regard to other physical modalities, combined therapy composed of ultrasound and interferential current has been studied in patients with fibromyalgia.^80^ Moretti et al. used 4000 Hz of current carrier, 100 Hz of amplitude-modulated frequency for interferential therapy and 1 MHz pulsed ultrasound at 20% of 2.5 W/cm².^80^ Patients were randomised in two groups; one group was treated once a week and the other was treated twice a week. Both groups showed improvement in visual analogue scale, tender points, Fibromyalgia Impact Questionnaire and Post Sleep Inventory. Yet, groups did not show difference in terms of generalised pain, quality of life and sleep quality (**Table 1**).^80^ The application of therapeutic heat/cold is a common technique used for painful musculoskeletal conditions. There are favourable results regarding the effectiveness of cold application and whole-body cryotherapy in fibromyalgia-related pain and/or quality of life.^81–83^ These results might be related to the anti-inflammatory effects of cold therapy, as there is cumulative evidence regarding the role of inflammation in fibromyalgia.^84^ In this regard, whole-body cryotherapy was suggested to modulate several neurotransmitters and cytokines, which might play role in pain alleviation.^85^ Klemm et al. evaluated whether clinical effects of whole-body cryotherapy in fibromyalgia could be explained by changes in cytokine levels. They found that IL-1, IL-6 and IL-10 levels were significantly altered over time. Moreover, patients with fibromyalgia demonstrated a significantly different response of IL-1, IL- 6 and IL- 10 to whole-body cryotherapy when compared to controls (**Table 1**).^85^ The use of extracorporeal shockwave therapy (ESWT) in fibromyalgia is controversial. Ramon et al. suggested the use of radial ESWT on each tender or trigger point for the treatment of the myofascial component of pain in fibromyalgia. Yet, they also recommended that a comprehensive supervised exercise should accompany the ESWT therapy.^86^

### The pathophysiological mechanisms by which physical modalities alleviate pain

Physical modalities can act as counterirritant stimulus, decrease muscle spasm, and reduce the nociceptive input. For instance, TENS and interferential current can temporarily reduce pain by serving as counterirritant stimuli, which correspond to the “gate control theory” of pain. This theory suggests that non-painful sensory inputs, such as electrical stimulation, can inhibit pain signals at the spinal cord level. Electrical muscle stimulation also leads to muscle contractions that may reduce spasms, improve muscle strength, and promote blood flow to the applied area. Superficial heat can temporarily reduce pain by acting as a counterirritant stimulus and increasing soft tissue extensibility. Physical modalities such as cryotherapy (cold packs, whole body cryotherapy) are thought to reduce inflammation. They promote local vasoconstriction and can thereby decrease inflammation. Phonophoresis, as a specific therapeutic approach, can facilitate the absorption of topical agents such as analgesic and/or anti-inflammatory gels; thus, can further alleviate pain and reduce inflammation when used with these agents.^87^

### Application of physical modalities and contraindications

Physical modalities are best used as adjunctive treatments and generally are not prescribed as stand-alone treatments. Instead, they usually accompany an exercise program, which is strongly recommended for the management of fibromyalgia by EULAR.^2, 88^ The application duration varies based on the type of physical modality applied. For example, TENS is generally used for 15–30 minutes per session.^87^ On the other hand, the duration is much less for LASER. There are several contraindications of physical modalities. Electrotherapeutic modalities such as TENS and interferential current are contraindicated in specific areas, including the anterior cervical region, heart, carotid sinuses, regions with anaesthesia, transthoracic area, and the abdomen of pregnant individuals. These treatments are also contraindicated for patients with a cardiac pacemaker, implanted defibrillator, other electrical devices, or those with venous/arterial thrombosis or thrombophlebitis. Physical modalities should not be applied over areas with malignancy, haemorrhage, infection, or ischemia, and on recently irradiated areas. Application of US on epiphyseal growth plates in skeletally immature patients, thrombotic regions, the eyes, gonads, and the spinal cord post-laminectomy is also contraindicated. Superficial heat is contraindicated in acute injuries/inflammation, haemorrhage, malignancy, impaired sensation, thrombophlebitis, or over the abdomen in pregnancy. Cold application is contraindicated in conditions like urticaria, cryoglobulinemia, Raynaud’s disease/phenomenon, paroxysmal cold haemoglobinuria, and areas with circulatory compromise, deep open wounds, or impaired somatosensory discrimination.^87^ The list can be extended depending on the physical modality applied.^87^

## CONCLUDING REMARKS AND FUTURE PERSPECTIVES

There are reports and growing evidence regarding the effectiveness of several physical modalities in fibromyalgia. Nevertheless, the evidence regarding many others is still scarce. In this regard, performing research focusing on the effectiveness and safety of physical modalities in patients with fibromyalgia would be of great value.

## CONFLICTS OF INTEREST

The author declares no conflicts of interest regarding the publication of this article.

## FUNDING INFORMATION

None to report.

## ETHICAL APPROVAL

This article does not contain any studies with human participants or animals performed by the author.

## DISCLAIMER

No part of this manuscript is copied or published elsewhere in whole or in part.
